# NT5E and FcGBP as key regulators of TGF-1-induced epithelial–mesenchymal transition (EMT) are associated with tumor progression and survival of patients with gallbladder cancer

**DOI:** 10.1007/s00441-013-1752-1

**Published:** 2013-12-06

**Authors:** Li Xiong, Yu Wen, Xiongying Miao, Zhulin Yang

**Affiliations:** Research Laboratory of Hepatobiliary Diseases, Second Xiangya Hospital, Central South University, 139# Middle Renmin road, Changsha, Hunan 410011 China

**Keywords:** FcGBP, Gallbladder cancer, NT5E, TGF-β1, Epithelial-mesenchymal transition

## Abstract

Epithelial–mesenchymal transitions (EMTs) are essential manifestations of epithelial cell plasticity during tumor progression. Transforming growth factor-β(TGF-β) modulates epithelial plasticity in tumor physiological contexts by inducing EMT, which is associated with the altered expression of genes. In the present study, we used DNA micro-array analysis to search for differentially expressed genes in the TGF-β1 induced gallbladder carcinoma cell line (GBC-SD cells), as compared with normal GBC-SD cells. We identified 225 differentially expressed genes, including 144 that were over-expressed and 81 that were under-expressed in the TGF-β1 induced GBC-SD cells. NT5E (CD73) is the most increased gene, while the Fc fragment of the IgG binding protein (FcGBP) is the most decreased gene. The expression patterns of these two genes in gallbladder adenocarcinoma and chronic cholecystitis tissue were consistent with the micro-array data. Immunochemistry and clinicopathological results showed that the expression of NT5E and FcGBP in gallbladder adenocarcinoma is an independent marker for evaluation of the disease progression, clinical biological behaviors and prognosis. The data from the current study indicate that differential NT5E and FcGBP expressions could be further evaluated as biomarkers for predicting survival of patients with gallbladder cancer and that NT5E and FcGBP could be promising targets in the control of gallbladder cancer progression.

**Electronic supplementary material:**

The online version of this article (doi:10.1007/s00441-013-1752-1) contains supplementary material, which is available to authorized users.

## Introduction

Gallbladder cancer (GBC) is the most common malignant tumor of the biliary tract, characterized by early lymph node invasion and distant metastases. It tends to be an aggressive tumor that spreads early and 90 % of GBC patients are presented at an advanced, inoperable stage (Husi and Grant 2001; Schrimpf et al. 2005). Most patients have died within the first year postoperatively. Many improvements have been made in diagnoses and treatments for GBC. However, the survival and outcome of most patients has not dramatically changed. Therefore it is very important to find new targets for the development of new therapeutic agents, as well as GBC biomarkers for monitoring both progression and treatment of the disease.

Genetic alterations resulting in altered mRNA levels have been described in GBC. Historically, a number of these genes have been identified, such as p53 tumor suppressor gene, *KRAS, BRAF, FHIT, CDKN2, HER2* and so on (Wistuba and Gazdar 2004). It has been reported that the expression level of Topoisomerase II was increased in GBC and vimentin was found to be significantly promoted in gallbladder carcinoma metastasis. Moreover, musashi-1, ALDH1, PEG10 and TSG101 were identified as carcinogenesis, progression and poor-prognosis-related biomarkers for gallbladder adenocarcinoma (Aebersold and Mann 2003; Li et al. 2004; Walikonis et al. 2000; Yoshimura et al. 2004). Due to its complicated background, it is very helpful to identify additional genes that may be up- or down-regulated in GBC, in order to better understand the process of metastasis and provide additional markers of diagnosis and treatments for the disease. As we all know, DNA microarray technology permits simultaneous analysis of the expression of large numbers of human genes (Mariadason et al. 2003). Furthermore, Chen et al. (2006) used DNA micro-arrays to identify potential markers for GBC and to examine the genetic differences between early and advanced GBC. When the GBC isolates were compared to the normal control samples, they identified 4,682 genes, including 2,270 that were over-expressed genes and 2,412 that were under-expressed genes in the GBC. Similarly in our previous studies, a total of 814 genes were overexpressed in the GBC cells revealed by DNA microarray technology, while 1,474 genes were underexpressed (data not shown). Even though so many differentially expressed genes were found, it would cost a great deal to screen out by conventional methods the valuable genes that are associated with the invasion and metastasis of GBC. In the present study, GBC-SD cells were treated with TGF-β1 to induce epithelial–mesenchymal transition (EMT) in order to specifically identify target genes of EMT during GBC. Theoretically, with TGF-β 1 induced EMT, a large number of genes unrelated to invasion and metastasis of GBC may be filtered and a designated study of the genes related to the mechanism of GBC invasion and metastasis can be carried out (Chen et al. 2006).

EMT is the transdifferentiation of epithelial cells into mesenchymal cells and this process has been shown to play an important role in the acquisition of metastatic phenotypes by carcinoma cells. Proteins inducing the occurrence of EMT in tumor cells are various, such as transforming growth factor-β TGF-β). TGF-β has a dual role in carcinogenesis. It can act as a tumor suppressor in early lesions, while contributing to the malignant progression by promoting invasion and metastasis in many advanced tumors (DeGiorgis et al. DeGiorgis et al. 2005; Stevens et al. 2003). Activation of TGF-β signaling is sufficient to induce EMT in cultured epithelial cells (Phillips et al. 2001).

NT5E is also called CD73 (ecto-5'-nucleotidase). It is a glycosyl phosphatidylinositol (GPI)-anchored purine salvage enzyme expressed on the surface of human T and B lymphocytes and catalyzes the final step in the hydrolysis of ATP to adenosine. It serves as a “master switch” between ATP-consuming and ATP-generating extracellular pathways (Collins and Grant 2007). However, NT5E has functions (such as an adhesive molecule in promoting tumor invasiveness in human glioblastoma) that are not related to its catalytic activity (Fukata et al. 2005; Vandenberghe et al. 2005). It has been found that CD73 is highly expressed in many human solid tumors and has participated in tumor neovascularization, invasiveness, metastasis and shorter life expectancy (Spychala 2000). In addition, NT5E expression and its generation of adenosine may relate to breast cancer progression and increased expression of CD73 in ER-negative cells may serve as a novel marker for more aggressive breast carcinoma (Kabbani et al. 2007; Kato et al. 2007; Rinner et al. 2007). Despite intensive efforts, the roles of NT5E in GBC have remained elusive.

FcGBP was first identified as an Fc portion of the IgG molecule binding site in intestinal and colonic epithelia, gall bladder, cystic duct, bronchus, submandibular gland, cervix uteri and in fluids secreted by these cells in humans. It might play a role in cell protection and anti-inflammation in tissues (Selbach and Mann 2006). O'Donovan et al. showed that FcGBP was differentially expressed in normal thyroid tissue, thyroid adenomas and thyroid carcinomas (Grant 2006). It has also been found that FcGBP expression was significantly reduced in both human and transgenic adenocarcinoma mouse prostate (TRAMP) prostate carcinomas (Witzmann et al. 2005). However, studies currently available in literature do not find FcGBP to have been involved in the pathogenesis of GBC.

In our research, DNA microarrays were applied to investigate the differentially expressed genes between GBC cells with TGF-β1 induced EMT and normal GBC cells. Then, an in-vivo study of gallbladder adenocarcinoma and chronic cholecystitis was performed to verify the results obtained in the microarray study and further investigate the role of NT5E and FcGBP in invasion and metastasis of gallbladder adenocarcinoma.

## Material and methods

### Cell culture

The human gallbladder cancer cell line GBC-SD was purchased from the Shanghai Cell Institute National Cell Bank (Shanghai, China). Cells were cultured in RPMI1640 medium (supplemented with 15 % fetal bovine serum (FBS) and 1 % penicillin/streptomycin) in a humidified incubator with 5 % carbon dioxide at 37 °C. To induce EMT, cells were cultured for 24 h and then incubated in media containing 10 μg/l of TGF-β1. Cells were collected for further analysis.

### Microarray procedures

Human 22 K Oligo Chips comprising 21,522 open reading frames were obtained from CapitalBio (Beijing, China). The cDNA probes obtained from GBC-SD cells were labeled with Cy5. An equal amount of cDNA probe obtained from TGF-β1 induced GBC-SD cells was labeled with Cy3. For hybridization, purified Cy3- and Cy5-labeled cDNA probes were mixed and applied to a microarray following incubation at 42 °C for 18 h under humid conditions. Fluorescent images of hybridized microarrays were scanned with a LuxScan -10KA device (CapitalBio, Beijing, China). Images were analyzed with LuxScan 3.0 (CapitalBio, Beijing, China) in accordance with the manufacturer’s instructions. DNA microarray overall linear calibration was made according to Cy5 and Cy3 and analysis was made of the fluorescent signal strength of Cy5 and Cy3; finally the ratio of Cy5/Cy3 was calculated. The Cy5/Cy3 ratio of a certain spot in the DNA microarray represented the expression abundance ratio of the mRNA between TGF-β1 induced GBC-SD cells and GBC-SD cells without TGF-β1 induction. Genes with weak fluorescent signal were deleted. The criteria of screening for differential expressed genes were as follows: upregulated genes with a ratio ≥ 2.0, downregulated genes with a ratio ≤ 0.5.

### RNA extraction and real-time polymerase chain reaction (PCR) analysis

Total RNA was extracted using RNAVzol (VIGOROUS, Beijing, China) and treated with DNase I (Invitrogen, USA). cDNA synthesis was performed using the cDNA Synthesis kit (Fermatas,USA), according to the manufacturer’s instructions.For the real-time PCR assay, cDNA was subjected to PCR amplification. Primers were designed to generate a PCR product less than 200 bp. The primer sequences were as follows: NT5E, forward:5'- TCGGGTTTTGAAATGGATAAAC -3' and reverse, 5'-TCAGGAATGCTGCTGTTTAGAA-3'. FCGBP, forward:5' - CCTATGGAGCTGGTGGATACTC -3' and reverse:5' - GCATAGTCAGAATGGATCACCA -3'. CDH1, forward:5' -CTGGACCATTCAGTACAACGACC-3' and reverse:5' -CTTCTCCGCCTCCTTCTTCATC-3', VIM, forward:5' -AATGACCGCTTCGCCAACTA-3' and reverse:5' -GCTCCTGGATTTCCTCTTCG-3', GAPDH, forward:5'-CAAGGTCATCCATGACAACTTTG-3 and reverse:5'-GTCCACCACCCTGTTGCTGTAG-3'. Thermal cycling conditions were: 94 °C for 2 min, followed by 30 cycles of 94 °C for 45 s, 55 °C for 30s and 72 °C for 10 min. Expression levels were normalized to GAPDH housekeeping gene. The visualized bands were quantified by BANDSCAN software. The average absolute intensity was presented as ± s. The statistical significance of differences was analyzed using Student’s *t*-test. *P* values < 0.05 were considered to be statistically significant.

### Western blotting

Total proteins extracted from GBC and chronic cholecystitis tissues were lysed with strong RIPA sample buffer (50 mmol/l Tris–HCl, pH 8.0, 150 mmmol/l NaCl, 1 mmol phenylmethylsulfonyl fluoride, 50 mmol/l DTT, and 1 % Triton X-100) on ice for 30 min. The supernatants were collected after centrifugation at 14,000 *g* at 4 °C for 10 min. After protein concentration was determined (Bradford Protein Assay kit, Beyotime Institute of Biotechnology,China), 50 μg proteins were mixed with 2 × SDS loading buffer and incubated at 100 °C for 10 min before loading and were subjected to electrophoresis in 11.5 % polyacrylamide gels. Proteins were electrotransferred onto PVDF membranes (Invitrogen, USA) in transfer buffer. Non-specific binding to the membrane was blocked for 1 h at room temperature with 5 % non-fat milk in tris buffer saline buffer (TBS) (20 mmol/l Tris–HCl, 150 mmol/l NaCl and 0.1 % Tween-20). Membranes were then incubated overnight at 4 °C with the following primary antibody: anti- NT5E (Santa Cruz, USA), anti-FcGBP (Santa Cruz, USA), followed by incubation with horseradish peroxidase-conjugated secondary antibody (Protintech, USA) respectively. Membranes were then washed with TBS buffer and signals visualized using the enhanced chemiluminescence system (Santa Cruz, USA). The visualized bands were quantified by BANDSCAN software. The signal intensity was normalized to β-actin. The average absolute intensity was presented as ± s. The statistical significance of differences was analyzed using Student’s *t*-test. *P* values < 0.05 were considered to be statistically significant.

### SiRNA and transfection

The three pairs of 19-nt siRNA duplexes targeting NT5E corresponding to the respective coding regions were designed and synthesized by GenePharma. NT5E siRNA-1 was raised against the sequence corresponding to nt 363–382 (5_-GACCAAAGCTGATCTCATA-3_), NT5E siRNA-2 corresponded to nt 792–811 (5_-GAAAGAAGAGGAAGATAAA-3_) and NT5E siRNA-3 corresponded to nt 1343–1362 (5_-CAGAAGACAAGGAGAATTA-3). All siRNA sequences were compared against human sequences deposited in GenBank^TM^ databases with BLAST (basic local alignment search tool) searches using NCBI (National Center for Biotechnology Information) tools.

For siRNA experiments, GBC-SD cells were grown overnight in 12-well plates. Cells were transfected with 100 pmol of siRNA using Lipofectamine^TM^ 2000 (Invitrogen) as instructed by the manufacturer. Forty-eight hours after transfection, the most effective recombinant vector was selected using a fluorescence microscope.

### Cell proliferation and apoptosis assays

GBC-SD cells transfected with siRNA specific to NT5E or with a nonspecific control siRNA were plated in triplicate in 96-well plates at a density of 1 × 10^5^ cells/well. Cell proliferation was analyzed by a methyl thiazolyl tetrazolium (MTT)-based assay. For each MTT assay, the medium in each well was replaced with 200 μl of medium containing 20 μl MTT. After 4 h of incubation, the MTT-containing medium was removed, 200 μl of DMSO was added to each well and the plate was agitated for 10 min in the dark to dissolve the MTT-formazan crystals. Sample absorbance was recorded at 570 nm. The above experiments were performed in triplicate and the results are presented as the mean ± SD.

### Transwell cell migration assay

Cell migration was assayed with 24-pore size Transwell migration chambers (Costar). Matrigel was thawed at 4 °C overnight and then diluted in RPMI1640 serum free-cold cell culture media. Then, 100 ul of the diluted matrigel was put into the upper chamber of a 24-well Transwell that was incubated at 37 °C for at least 4 to 5 h for gelling. GBC-SD cells transfected with siRNA specific to NT5E or with a nonspecific control siRNA were digested by Trypsin/EDTA, washed 3 times with RPMI1640 containing 1 % FBS and resuspended in media containing 1 % FBS at a density of 106 cells/ml. Then, 100 ul of the cell suspension was put onto the matrigel.The lower chamber of the transwell was filled with 600 ul of culture media containing 5 ug/ml fibronectin, as an adhesive subtrate. After being incubated at 37 °C for 24 h, transwells were removed from 24-well plates and stained with Diff-Quick solution (Fischer Scientific). Non-migrated cells were removed using a cotton swab and the migrated cells were then photographed using a microscope (MZ16FA; Leica) equipped with a digital camera (DC 500; Leica). To quantify the number of migrated cells, stained cells were subsequently extracted and detected on a standard microplate reader at 560 nm (Spectra-MAX190; Molecular Devices).

### Wound healing assay

GBC-SD cells transfected with siRNA specific to NT5E or with a nonspecific control siRNA were detached by trypsin, resuspended in RPMI1640 medium and plated in triplicate at 3 × 105 cells per well of a 24-well plate. After the cell became confluent, cells were starved in a RPMI serum-free medium for 24 hrs; then one artificial wound per well was scratched into the monolayers with a sterile plastic 10-μl micropipette tip to generate a uniform wound. After wounding, the culture medium was removed and cells were washed at least twice in PBS. Wound closure was monitored by digital photographs taken in a phase-contrast microscope (Nikon Diaphot 300, Tokyo, Japan) across the wound at the moment of wounding and at 24 hrs post-wounding.

### Case selection

Samples of 108 patients who underwent surgical resection or biopsy from 1996 to 2011 were identified as having gallbladder adenocarcinoma. Twetny-four of the patients (22.2 %) were male and 84 patients (77.8 %) were female, with an average age of 54.5 ± 10.6 years. All diagnoses were based on morphological criteria, immunohistochemical staining and clinical findings. The histopathologic subtypes of the 108 adenocarcinomas were as follows: 36 cases (33.3 %) of well-differentiated adenocarcinoma, 31 cases (28.7 %) of moderately-differentiated adenocarcinoma, 30 cases (27.8 %) of poorly-differentiated adenocarcinoma and 11 cases (10.2 %) of mucinous adenocarcinoma. Fifty-nine patients (55.0 %) had regional lymph node metastasis, in 58 patients (54.0 %) accompanied by gallstones. Surgery included radical resection for 32 adenocarcinomas (32.0 %), palliative surgery for 44 adenocarcinomas (44.0 %) and biopsy for 24 cases (24.0 %) without resection. We selected 46 peritumoral tissues (distance from cancer >3 mm) from 108 gallbladder adenocarcinoma tissues. Among the 46 peritumoral tissues, ten tissues were normal, one tissue showed mild dysplasia, 12 tissues showed moderate dysplasia and 14 tissues showed severe dysplasia.

From 1996 to 2011, we gathered other specimens of 80 patients with cholecystectomy diagnosed as adenoma (30), poly (15), simple chronic cholecystitis (15), or chronic cholecystitis with stones (20). General data were as follows:

- There were 12 male patients (40.0 %) and 18 female ones (60.0 %) in the adenoma group, with an average age of 48.5 ± 9.4, maximum diameter 0.9–2.2 cm. Pathological findings revealed normal epithelia in five cases mild dysplasia in ten cases, moderate dysplasia in nine cases and severe dysplasia in six cases.
- There were five male patients (33.3 %) and ten female ones (66.7 %) among the poly group, with an average age of 50.8 ± 9.6, maximum diameter 0.8–0.15 cm. Pathological findings revealed adenomatous polyp, among which ten cases were normal epithelia to mild dysplasia and five cases were moderate dysplasia to severe dysplasia.
- There were 15 male patients (42.9 %) and 20 female ones (57.1 %) in the chronic cholecystitis groups, with an average age of 43.2 ± 12.4, maximum diameter 0.9–2.2 cm. Pathological findings revealed normal epithelia in 11 cases, mild dysplasia in 12 cases, moderate dysplasia in seven cases and severe dysplasia in five cases.

The specimens were prepared as paraffin-embedded tissues and then 4 μm sections were made for further study. This study was carried out in accordance with the Declaration of Helsinki (2000) of the World Medical Association and it was approved ethically by the Second Xiangya Hospital attached to Central South University. Informed written consent forms were obtained from all subjects who participated in the study.

### EnVision™ immunohistochemistry

An EnVision™ Detection Kit was purchased from Dako Laboratories (Carpinteria, CA, USA). 4-μm sections were cut from routinely paraffin-embedded tissues, deparaffinized and incubated with 3 % H_2_O_2_ in the dark for 15 minutes, then digested by EDTA-trypsin for 15 minutes. The sections were incubated with primary antibody (anti- NT5E and anti-FcGBP) for 60 minutes, then second antibody for 30 min after being incubated 3 times with PBS. Solution A was added to the sections for 30 minutes. After DAB staining and haematoxylin counter-staining, the slides were dehydrated with alcohol (70–100 % concentration) and then incubated 3 times in xylene. Finally, neutral balsam was mounted with the slides. Ten random fields were examined per section. The percent of positively stained cells relative to the total number of cells was counted. The strength of staining was rated on a scale of 1 to 3. A score of 1 represents little to no positive staining or uncertain weak staining; 2 represents weak to moderate staining; 3 represents moderate to strong staining. A section was determined as positive for NT5E or FcGBP when the percent of positively stained cells was >10 % and staining strength >2. The few sections where percent positive staining was 5 % to 10 % and staining strength was 3 were also regarded as positive.

### Statistical analysis

Data were analyzed using SPSS 13.0. The inter-relationship NT5E or FcGBP expression with histology or clinical factors was analyzed using χ^2^ or Fisher’s exact test. Kaplan–Meier and time series test (log-rank test) were used for univariate survival analysis. The Cox proportional hazards model was used for multivariate analysis and to determine the 95 % confidence interval.

## Results

### Identification of genes differentially expressed in the TGF-β1 induced GBC-SD cells and normal GBC-SD cells

**Fig. 1:**
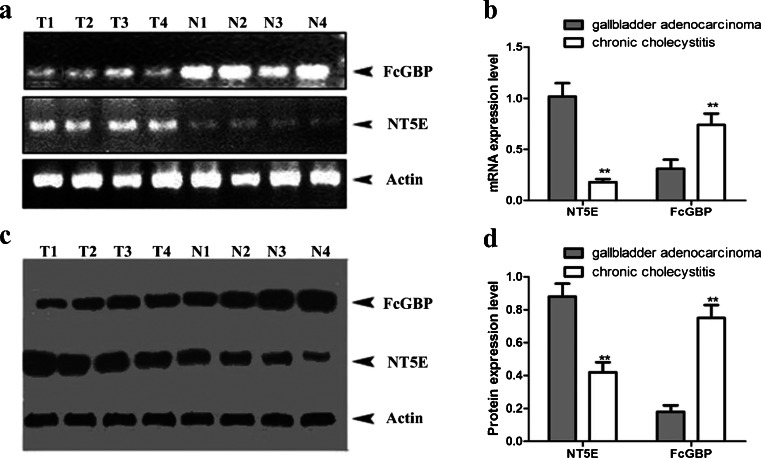
RT-PCR and Western blot analysis of the expression level of NT5E and FcGBP in the gallbladder adenocarcinoma and chronic cholecystitis samples. **a**, **b** The total RNA was both extracted from specimens of 15 patients diagnosed as having gallbladder adenocarcinoma and 15 patients diagnosed as having chronic cholecystitis. The mRNA transcription expression levels of NT5E and FcGBP were evaluated by RT-PCR. The values were normalized to GAPDH as an internal control. **c**, **d** Western blotting analysis of the levels of NT5E and FcGBP in the gallbladder adenocarcinoma and chronic cholecystitis samples. Proteins were separated by 11.5 % SDS-PAGE. The gels were electroeluted to PVDF membrane and each blot was analyzed with a different antibody: anti- NT5E and anti-FcGBP. The values were normalized to actin as an internal control. Mean expression values ± SE are shown. *Asterisks* indicate that the expression differences between TGF-β1 induced GBC-SD cells and normal GBC-SD cells are statistically significant (*p* < 0.05 for Student’s *t*-test)

To explore the mechanism of invasion and metastasis of gallbladder cancer, DNA microarray technology was applied to investigate the differentially expressed genes between GBC cells with TGF-β1 induced epithelial mesenchymal transition (EMT) and normal GBC cells. Results revealed by DNA microarray showed that Hex, interior label and external label were normal in positive control and negative in negative control, with an authentic repetitiveness of housekeeping genes (ratio CV ≤ 0.3) and leak rate ≤3‰, indicating that the data are valid without genomic DNA contamination. The distribution of differential gene expressions in TGF-β1 induced and control GBC-SD cells is shown in a scatter plot form in Fig. 1. These items suggested that the samples were not pollution. Two hundred and twenty-five differentially expressed genes were identified between GBC-SD cells with and without TGF-β1 induced EMT, among which 144 genes were up-regulated (Supplement Table 1) and 81 genes were down-regulated (Supplement Table 2) in GBC cells after TGF-β1 induced EMT. NT5E is the most increased gene while FcGBP is the most decreased gene.

After that, we performed further gene ontology (GO), pathway and bioinformatics analyses using the molecule annotation system (MAS 2.0). A hypergeometric distribution was applied for the *P*-value in GO and pathway investigations. According to GO classification, differentially expressed genes were grouped into the pathway of oxidation, protein binding and cell adhesion (Supplement Fig 2). Most interestingly, revealed by the pathway analysis, these genes were associated with the cyclin dependent kinase pathway regulation, intrinsic prothrombin activation pathway, cell cycle regulation, phosphoinositide-mediated signaling, etc. Through the analysis and comparison of the gene expression profile, NT5E and FcGBP were new differentially expressed genes during TGF-β1 induced EMT. After reference review, we thought that NT5E and FcGBP might play important roles in the invasion and metastasis of gallbladder cancer with TGF-β1 induced EMT. So these two genes were chosen for further research.

### Validation of the microarray data by RT-PCR and Western blot

The in-vivo study of gallbladder adenocarcinoma and chronic cholecystitis was performed to verify the expression levels of NT5E and FcGBP in invasion and metastasis of gallbladder adenocarcinoma. The total RNA and protein were both extracted from specimens from 15 patients diagnosed as having gallbladder adenocarcinoma and 15 patients diagnosed as having chronic cholecystitis. The mRNA transcription and protein expression levels of NT5E and FcGBP were evaluated by RT-PCR and Western blot respectively. Figure 1 shows that NT5E mRNA was significantly lower in chronic cholecystitis than in gallbladder adenocarcinoma (0.225 ± 0.021 vs 1.012 ± 0.019, *P* < 0.01), while FcGBP mRNA was significantly higher in chronic cholecystitis than in gallbladder adenocarcinoma (0.321 ± 0.017 vs 0.078 ± 0.020, *P* < 0.01). Western blot demonstrated that NT5E expression was significantly higher in gallbladder adenocarcinoma than in chronic cholecystitis (0.826 ± 0.011 vs 0.412 ± 0.017, *P* < 0.01), while the expression of FcGBP protein was significantly lower in gallbladder adenocarcinoma than in chronic cholecystitis (0.084 ± 0.017 vs 0.187 ± 0.025, *P* < 0.01). NT5E expression was upregulated and FcGBP expression was downregulated in gallbladder adenocarcinoma compared to chronic cholecystitis in vivo, which was consistent with the DNA microarray data (Fig. 1).

### NT5E regulates the expression of EMT-associated gene in GBC-SD cells

**Fig. 2:**
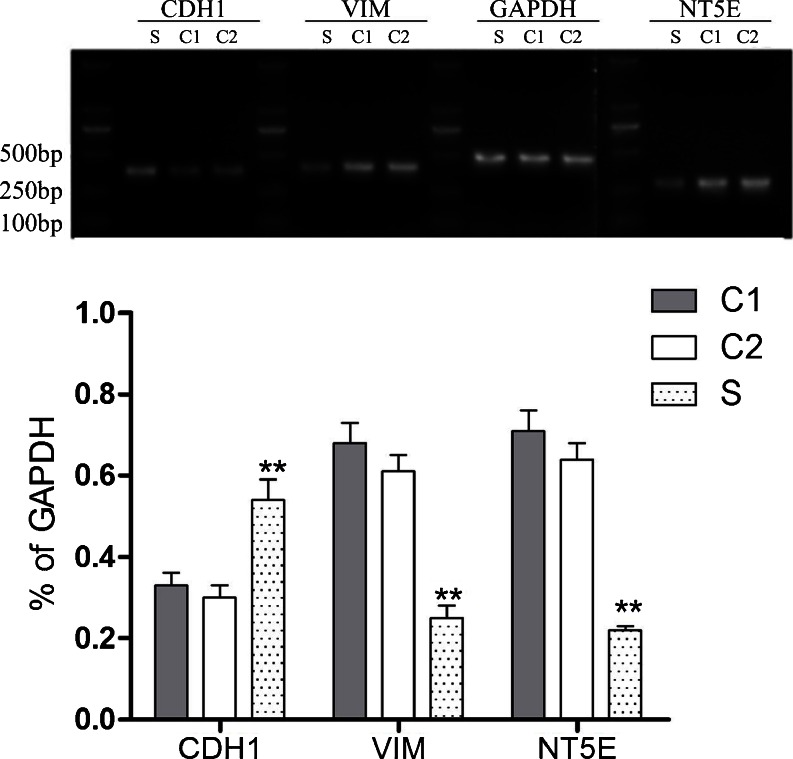
Transfected with pRNA-siNT5E-1 induced an increase in VIM mRNA and a decrease in CDH1 mRNA in GBC-SD cells. *S*: GBC-SD cells transfected with pRNA-siNT5E-1; *C1*: GBC-SD cells transfected with unrelated sequence vector; *C2*: non-transfected group. NT5E and VIM gene (^**^ *P* < 0.01 compared to transfected with unrelated sequence vector or non-transfected group)

We examined the effect of NT5E on the expression of key regulators of EMT including CDH1 and VIM. Real-time PCR analysis indicated that the expression of NT5E and VIM gene in the GBC-SD cells transfected with pRNA-siNT5E-1 was significantly lower than in the unrelated sequence vector transfected group and non-transfected group (*P* < 0.01), while the expression level of CDH1 gene in the GBC-SD cells transfected with the pRNA-siNT5E-1 vector was significantly higher than others (*P* < 0.01), as shown in Fig. 2. The expression of NT5E , CDH1 and VIM mRNA had no statistical significance between the group that was transfected with the unrelated sequence vector and the non-transfected group (*P* > 0.05).

### Down-regulation of NT5E affects cell viability

**Fig. 3:**
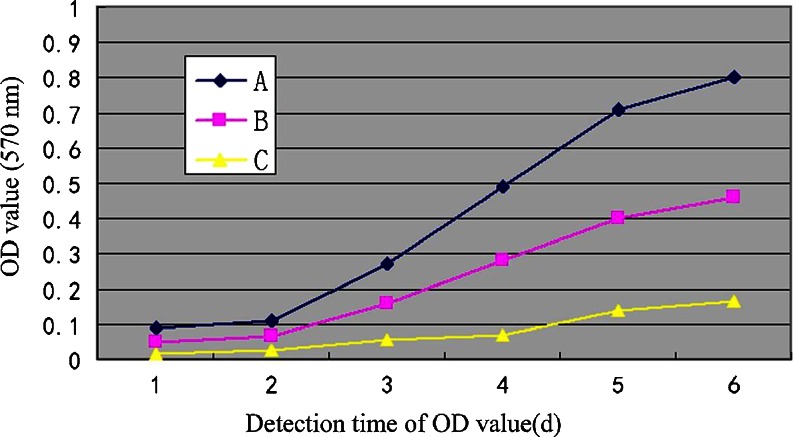
Effects of NT5E knockdown using NT5E siRNA on regulation of gallbladder cancer cell viability. Gallbladder cancer cells were grown and transfected with NT5E siRNA for 48 h and subjected to cell viability MTT assay. Data are shown as means ± SEM (*n* = 6)

To determine whether siRNA-mediated knockdown of NT5E affected cell viability, an MTT assay was performed to investigate cellular proliferation. Figure 3 shows that the proliferation of GBC-SD cells transfected with pRNA- siNT5E-1 was slower than that of the unrelated sequence vector group and non-transfected group cells from the third day and continued to the sixth day (*P* < 0.05). We determined that the proliferation of GBC-SD cells transfected with the unrelated sequence vector and non-transfected had no statistical significance all the time (*P* > 0.05).

### NT5E regulates cell migration

**Fig. 4:**
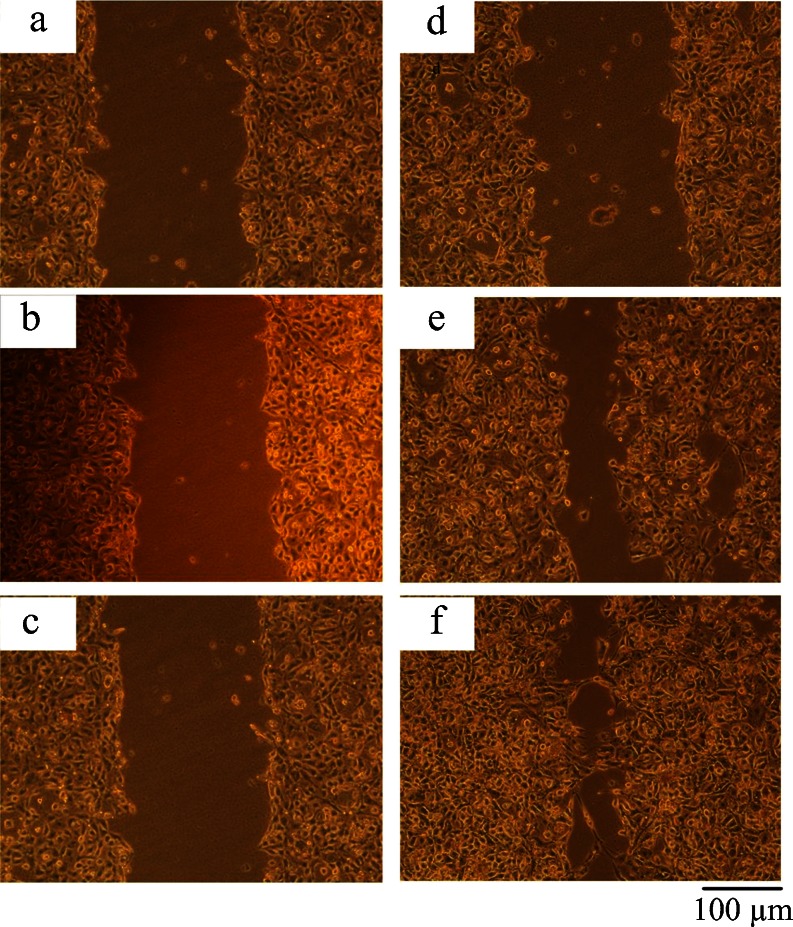
siRNA-mediated knockdown of NT5E affected cell migration in vitro by scratch wound healing assay. **a**, **d** GBC-SD cells transfected with pRNA-siNT5E-1. **b**, **e** GBC-SD cells transfected with unrelated sequence vector. **c**, **f** Non-transfected group. **a**, **b**, **c** Wound at time 0 (0 h) for GBC-SD cells incubated with control, serum-free media. **d**, **e**, **f** 24 hrs after wounding for SW756 cells incubated with control, serum-free media. ^**^ *P* < 0.01 compared to transfected with unrelated sequence vector or non-transfected group. *Scale bar* 100 μm

The role of NT5E in regulating cell migration was also examined. The directed migration of GBC-SD cells transfected with pRNA- siNT5E-1, unrelated sequence vector transfected group and non-transfected group cells was compared using an in-vitro wound assay. In-vitro wound healing assays have been used with multiple cell types and are a classic and commonly used method for studying cell migration and the biology underlying it. As shown in Fig. 4, when a confluent plate of cells was scratched by a sterile pipette tip, the areas of the scratch in three groups were the same at 0 hour. After 48 hours, a small number of the GBC-SD cells transfected with pRNA -siNT5E-1 migrated to the scratch area, while the cells transfected with unrelated sequence vector pRNAi-NC and non-transfected cells migrated obviously.

**Fig. 5:**
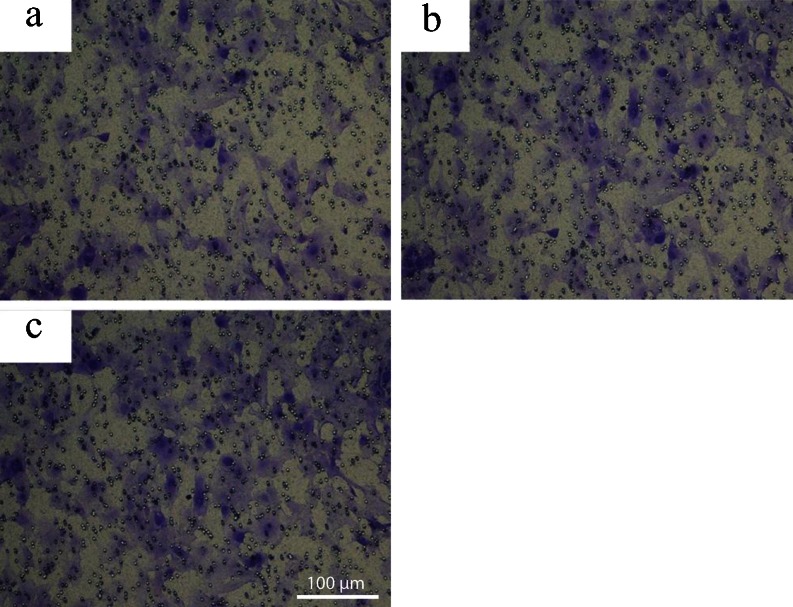
siRNA-mediated knockdown of NT5E affected cell migration by Transwell assay. **a** GBC-SD cells transfected with pRNA-siNT5E-1. **b** GBC-SD cells transfected with unrelated sequence vector. **c** Non-transfected group. Cell numbers in three microscope fields (×200) from each Transwell were counted. Columns, mean of migrating cells done in triplicate; ^**^ *P* < 0.01 compared to transfected with unrelated sequence vector or non-transfected group. *Bars* 100 μm

To further confirm the role of NT5E in regulating cell motility, a transwell chamber invasion assay was used. Consistent with the results from the wound assay, Fig. 5 shows that the number of GBC-SD cells that were transfected with pRNA -siNT5E-1 that passed through the membrane was lower than that of the unrelated sequence vector transfected group and non-transfected GBC-SD cells; the difference was statistically significant (*P* < 0.05), while there was no statistically significant difference between the unrelated sequence vector transfected group and non-transfected group cells (*P* > 0.05).

### Differential expression of NT5E and FcGBP and their clinicopathological significance in benign and malignant lesions of the gallbladder

The results (Supplement Table 3) show that NT5E expression was significantly more up-regulated in gallbladder adenocarcinoma (54.6 %) than in peritumoral tissues (30.4 %) and other benign lesions including adenoma (16.7 %), polyps (13.3 %) and chronic cholecystitis (11.4 %) (*P* < 0.01). The ratio of positive FcGBP expression was significantly down-regulated in gallbladder adenocarcinoma (48.1 %) than in peritumoral tissues (76.1 %), adenoma (80.0 %), polyps (86.7 %) and chromic cholecystitis (85.7 %) (*P* < 0.05) (Supplement Table 3). An atypical hyperplasia in gallbladder epithelium was observed in the benign lesions with NT5E positive-expression and/or FcGBP negative-expression.

**Fig. 6:**
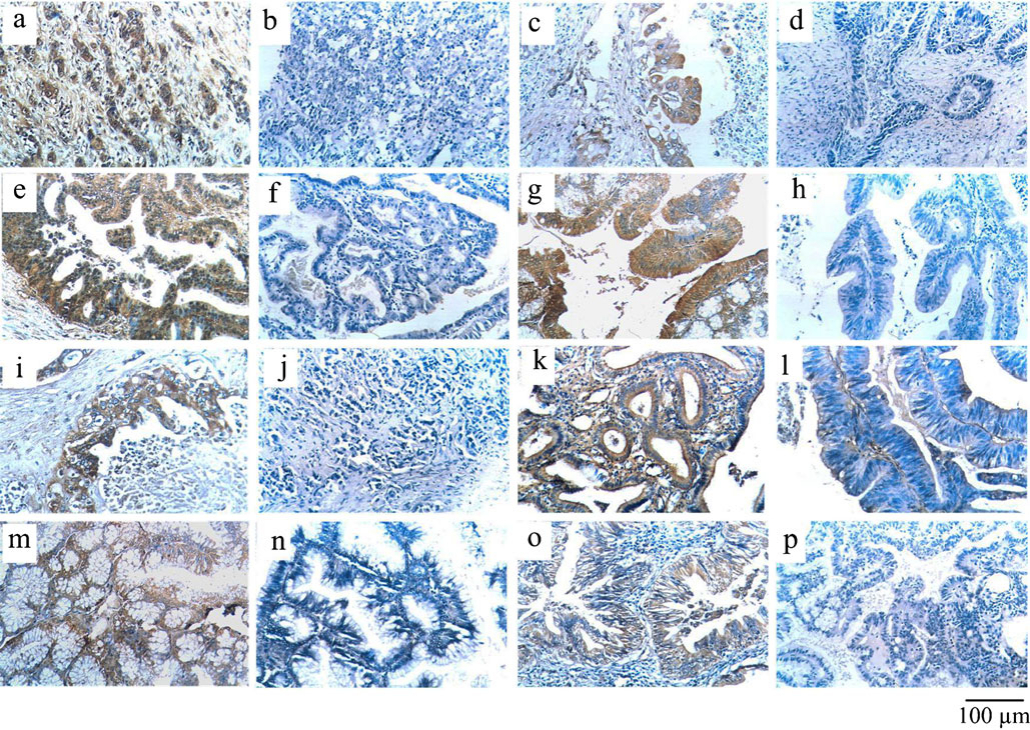
NT5E and FcGBP expression in benign and malignant lesions of gallbladder using EnVision immunohistochemistry (original magnification ×200). NT5E and FcGBP positive reactions were mainly localized in the cytoplasm. **a** Poorly differentiated gallbladder adenocarcinoma, NT5E(+). **b** Poorly differentiated gallbladder adenocarcinoma NT5E (−). **c** Adjacent tissues to GBC with severe atypical hyperplasia NT5E(+). **d** Adjacent tissues to GBC with severe atypical hyperplasia NT5E(−). **e** Adenoma with severe atypical hyperplasia NT5E(+). (**f**) Adenoma with moderate atypical hyperplasia NT5E (−). **g** Polyp with moderate atypical hyperplasia NT5E(+). **h** Polyp with mild atypical hyperplasia NT5E(−). **i** Moderately differentiated gallbladder adenocarcinoma FcGBP(+). **j** Poorly differentiated gallbladder adenocarcinoma FcGBP(−). **k** Adjacent tissues to GBC with mild atypical hyperplasia FcGBP(+). **l** Adjacent tissues to GBC with severe atypical hyperplasia FcGBP(−). **m** Polyp with mild atypical hyperplasia FcGBP(+). **n** Polyp with moderate atypical hyperplasia FcGBP(−). **o** Polyp with moderate atypical hyperplasia FcGBP(+). **p** Polyp with severe atypical hyperplasia FcGBP(−)

**Fig. 7:**
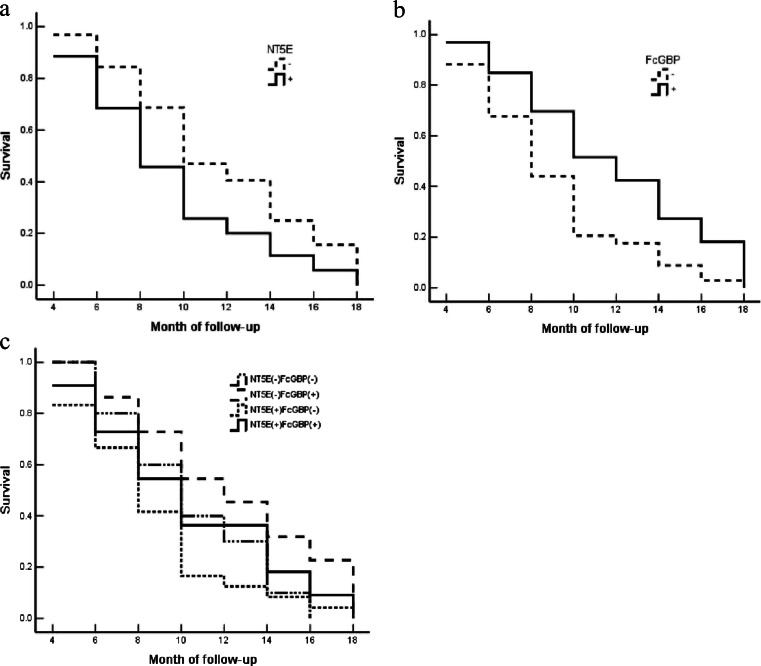
NT5E and FcGBP expression and survival in patients with adenocarcinoma of gallbladder. **a** Kaplan–Meier plots of overall survival in patients with gallbladder adenocarcinoma and with NT5E positive and negative expression. **b** Kaplan–Meier plots of overall survival in patients with gallbladder adenocarcinoma and with FcGBP positive and negative expression. **c** Kaplan–Meier plots of overall survival in patients with gallbladder adenocarcinoma and with NT5E (+)(−) and FcGBP (+)(−) expression

The NT5E expression in well-differentiated adenocarcinoma, with small tumor size (diameter < 2 cm), no lymph node metastasis and no peripheral tissue invasion was significantly down-regulated compared to that in poor-differentiated adenocarcinoma, with larger tumor size (diameter ≥ 2 cm), lymph node metastasis and peripheral tissue invasion (*P* < 0.5 or *P* < 0.01) (Fig. 5 and Supplement Table 4). However, the expression of FcGBP showed a negative correlation in those cases (*P* < 0.05 or *P* < 0.01) (Fig. 6 and Supplement Table 4). The monovariable Kaplan–Meier survival analysis showed that the pathological type, tumor maximum diameter, lymph node metastasis, peripheral tissue invasion, NT5E and FcGBP expression were significantly related to the average survival time of patients with the gallbladder adenocarcinoma (*P* < 0.05 or *P* < 0.01) (Supplement Table 5). Furthermore, the survival time of patients with NT5E expression was significantly shorter than those without NT5E expression (*P* < 0.05) (Fig. 7a). In addition, FcGBP showed a negative correlation in those cases (*P* < 0.05) (Fig. 7b). Interestingly, the overall survival time of cases with NT5E(−)FcGBP(+) was significantly longer than those with NT5E(+)FcGBP(−) (Fig. 7c). Multivariable Cox analysis revealed that NT5E expression (*P* = 0.019) or FcGBP expression (*P* = 0.004) was negatively associated with survival period and positively associated with mortality after surgery (Supplement Table 6).

These results showed that the expression of NT5E and FcGBP in gallbladder adenocarcinoma is an independent marker for evaluation of the disease progression, clinical biological behaviors and prognosis. NT5E and FcGBP might have an important effect on the regulated EMT in malignant lesions (including GBC). Patients with FcGBP expression had a better prognosis, while patients with NT5E expression had a worse prognosis.

## Discussion

Gallbladder cancer tends to be the most common malignant tumor observed in Chile, Japan, and northern India. The main cause of death in patients is the invasion and metastasis of cancer. Cancer is a genetic disease that involves cumulative genetic and epigenetic alterations, including the activation of oncogenes and the inactivation of tumor suppressor genes. So gene expression profiling analysis in cancer can shed light on the mechanisms and pathways that regulate the transition from normal growth to malignant proliferation. However, only limited information is currently available concerning the genetic mechanisms that are involved in the malignant transformation of gallbladder cancer. In the present research, a DNA microarray was used to identify the differentially expressed genes in TGF-β1 induced GBC-SD cells, as compared with normal GBC-SD cells. As we know, epithelial–mesenchymal transition (EMT) is a key event in tumor invasion (Dosemeci et al. 2007) and TGF-β1 is an important inducer of EMT in cancer progression (McClatchy et al. 2007). With TGF-β1 induced EMT, a large number of genes unrelated to invasion and metastasis of GBC were filtered and a designated study of the genes related to the mechanism of GBC invasion and metastasis was carried out in the present study.

Future design of disease process-specific interventions, such as prevention of tumor progression, will to a large extent depend on knowledge of mechanisms that determine biological specificity of TGF-b actions. We demonstrate that functional genomic approaches provide a new tool to define roles of critical nodes in TGF-b’s signaling circuitry, based on inspection of their proximal signaling targets in the context of well-defined physiological responses. For example, we show that in the context of TGF-b-induced EMT, 144 upregulated genes and 81 downregulated genes were identified using the DNA microarray strategy. NT5E is the most increased gene, while FCGBP is the most decreased gene in the TGF-β1 induced GBC-SD cells, which suggest that these two genes may play important roles in EMT.

Our research found the decreased expression of NT5E and over-expression of FcGBP in the TGF-β1 induced GBC-SD cells. On the basis of the dramatic fold changes, NT5E and FCGBP were selected and then reexamined by the use of RT-PCR and Western blot, which employed specimens of patients diagnosed as having gallbladder adenocarcinoma and chronic cholecystitis. The expression pattern of these genes in the tissues was consistent with the microarray data. We further examined the expression patterns of NT5E and FcGBP in benign and malignant lesions of gallbladder by immunochemistry and the clinicopathological significance between them is also evaluated. The elevated expression of NT5E and decreased expression of FcGBP in gallbladder adenocarcinoma suggested that they are important markers for progression, clinical biological behavior and prognosis. Our results also found that patients with high FcGBP and low NT5E expression in their tumors are more likely to suffer from invasion and metastatic recurrence. The monovariable Kaplan–Meier survival analysis suggested that NT5E and FcGBP expression were significantly related to the average survival time of patients with gallbladder adenocarcinoma. Multivariable Cox analysis revealed that NT5E positive-expression or FcGBP negative-expression was negatively associated with survival period. So measurement of NT5E and FcGBP expression could be used as a tool for early detection of gallbladder cancer in benign lesions as well as population screening. These patients may need to be monitored closely for clinical signs of relapse. Furthermore, we wanted to demonstrate whether NT5E participated in EMT regulation or not. The expression of NT5E gene in GBC-SD cells was significantly inhibited after being transfected with interference vector pRNA-siNT5E with the up-regulation of CDH1 gene and down-regulation of VIM gene. This is maybe related to the process of epithelial–mesenchymal transition of GBC-SD cells. We found decreased expression of NT5E and overexpression of FcGBP in malignant gallbladder tissues compared to normal and benign gallbladder tissues.

However, our current study is a promising proof-of-principle study. Future studies will confirm our data using a larger sample size before they can be useful as biomarkers for the prediction of survival of gallbladder cancer patients. In addition, more mechanistic studies will be needed to understand their role in regulation of gallbladder cancer progression and metastasis.

## Electronic supplementary materials

Below is the link to the electronic supplementary material.

Supplement Figure 1 (DOC 47 kb)

Supplement Figure 2 (DOC 84 kb)

Supplement Table 1 (DOC 175 kb)

Supplement Table 2 (DOC 89 kb)

Supplement Table 3 (DOC 29 kb)

Supplement Table 4 (DOC 58 kb)

Supplement Table 5 (DOC 33 kb)

Supplement Table 6 (DOC 29 kb)

Supplement Table 1-3 (DOC 32 kb)

Supplement Table 1-4 (DOC 32 kb)

Supplement Table 1-5 (DOC 32 kb)
