# Supplementary material for: NT5E and FcGBP as key regulators of TGF-1-induced epithelial–mesenchymal transition (EMT) are associated with tumor progression and survival of patients with gallbladder cancer
Source: Cell Tissue Res. 2013 Dec 6;355(2):365–74. doi: 10.1007/s00441-013-1752-1 (PMC3921456; doi:10.1007/s00441-013-1752-1)
Supplement: Supplementary file 1 — (DOC 47 kb) [file 441_2013_1752_MOESM1_ESM.doc]

**Figure legends**


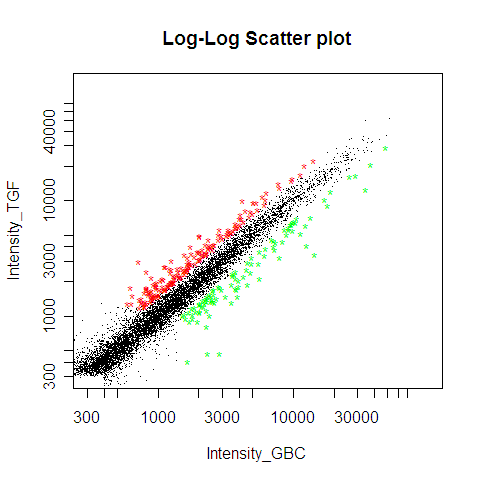


**Supplement Figure 1.** Scatter plot of differentiated expressed genes in TGF-β1-treated gallbladder cancer cells. Total RNA from TGF-β1-treated and control gallbladder cancer cells was isolated for cDNA microarray analysis of differentiated expressed genes. The scanned data are plotted for the comparative scatter plot analysis. The X or Y axis is fluorescence signal strength value of these two samples, respectively. Each data point on the graph represents a particular gene chip hybridization signal as red and green data points are the Ratio value of GBC - SD (bigger than 1.5) and TGF-β1-induced GBC–SD (less than 0.67), respectively. The differentiated expressed genes, marked as black points, presented the ratio value (between 0.5 to 2) of GBC-SD/TGF-β1-induced GBC-SD. A total of 264 differentiated expressed genes between TGF-β1-induced and control GBC-SD cells, among which 166 genes were upregulated, whereas 98 genes were downregulated.
