# Supplementary material for: NT5E and FcGBP as key regulators of TGF-1-induced epithelial–mesenchymal transition (EMT) are associated with tumor progression and survival of patients with gallbladder cancer
Source: Cell Tissue Res. 2013 Dec 6;355(2):365–74. doi: 10.1007/s00441-013-1752-1 (PMC3921456; doi:10.1007/s00441-013-1752-1)
Supplement: Supplementary file 2 — (DOC 84 kb) [file 441_2013_1752_MOESM2_ESM.doc]

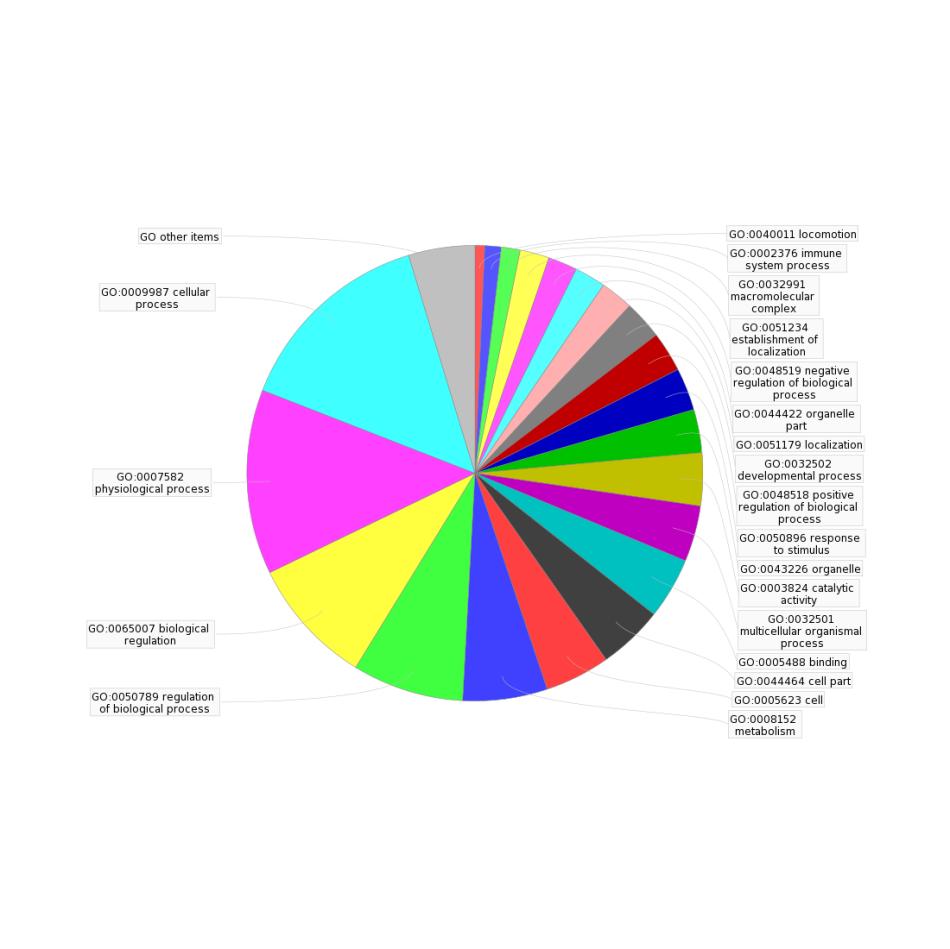


**Supplement Figure 2.** GO analysis of biological functions of these differentiated expressed genes in TGF-β1-treated gallbladder cancer cells.
