# Supplementary material for: NT5E and FcGBP as key regulators of TGF-1-induced epithelial–mesenchymal transition (EMT) are associated with tumor progression and survival of patients with gallbladder cancer
Source: Cell Tissue Res. 2013 Dec 6;355(2):365–74. doi: 10.1007/s00441-013-1752-1 (PMC3921456; doi:10.1007/s00441-013-1752-1)
Supplement: Supplementary file 3 — (DOC 175 kb) [file 441_2013_1752_MOESM3_ESM.doc]

**Supplement Table 1. Overexpressed genes in TGF-β1-induced GBC-SD cells vs. those in control GBC-SD cells**

| **Associated Gene Name** | **Description** | **Cy5 Intensity (GBC)** | **Cy3 Intensity (TGF)** | **Ratio** |
| --- | --- | --- | --- | --- |
| NRM | nurim (nuclear envelope membrane protein) | 1479 | 2578 | 1.5013 |
| APOBEC3D | apolipoprotein B mRNA editing enzyme, catalytic polypeptide-like 3D | 1001 | 1741 | 1.5015 |
| NUP62CL | nucleoporin 62kDa C-terminal like | 2517 | 4299 | 1.5024 |
| BRD8 | bromodomain containing 8 | 1428 | 2603 | 1.5041 |
| DSCC1 | defective in sister chromatid cohesion 1 homolog | 1558 | 2897 | 1.5056 |
| HSP90AA1 | heat shock protein 90kDa alpha (cytosolic), class A member 1 | 11241 | 18458 | 1.5100 |
| EIF2C1 | eukaryotic translation initiation factor 2C, 1 | 1437 | 2481 | 1.5105 |
| NCAPG2 | non-SMC condensin II complex, subunit G2 | 1292 | 2272 | 1.5132 |
| MDC1 | mediator of DNA-damage checkpoint 1 | 1134 | 1955 | 1.5133 |
| PRKDC | protein kinase, DNA-activated, catalytic polypeptide | 6139 | 11592 | 1.5160 |
| NUCKS1 | nuclear casein kinase and cyclin-dependent kinase substrate 1 | 3924 | 7017 | 1.5163 |
| EIF4A2 | eukaryotic translation initiation factor 4A2 | 3394 | 5920 | 1.5169 |
| ITGB3BP | integrin beta 3 binding protein (beta3-endonexin) | 912 | 1587 | 1.5185 |
| MLF1IP | MLF1 interacting protein | 1615 | 3024 | 1.5195 |
| WDR54 | WD repeat domain 54 | 1191 | 2121 | 1.5199 |
| P4HA2 | prolyl 4-hydroxylase, alpha polypeptide II | 1141 | 2308 | 1.5211 |
| TOPBP1 | topoisomerase (DNA) II binding protein 1 | 1444 | 2650 | 1.5218 |
| RNF141 | ring finger protein 141 | 1279 | 2213 | 1.5241 |
| NCAPH | non-SMC condensin I complex, subunit H | 1653 | 2921 | 1.5249 |
| NUF2 | NUF2, NDC80 kinetochore complex component | 2273 | 4216 | 1.5290 |
| SSRP1 | structure specific recognition protein 1 | 4750 | 7756 | 1.5290 |
| CCDC88A | coiled-coil domain containing 88A | 877 | 1608 | 1.5305 |
| RFC4 | replication factor C (activator 1) 4, 37kDa | 2017 | 3651 | 1.5311 |
| CENPA | centromere protein A | 1653 | 2876 | 1.5316 |
| FEN1 | flap structure-specific endonuclease 1 | 2063 | 3815 | 1.5341 |
| FAR1 | fatty acyl CoA reductase 1 | 786 | 1521 | 1.5341 |
| TRMT5 | TRM5 tRNA methyltransferase 5 homolog | 9735 | 15060 | 1.5352 |
| IFI27L2 | interferon, alpha-inducible protein 27-like 2 | 3473 | 5824 | 1.5392 |
| MATR3 | matrin 3 | 3612 | 5670 | 1.5405 |
| CCDC14 | coiled-coil domain containing 14 | 3216 | 5440 | 1.5439 |
| BLM | Bloom syndrome, RecQ helicase-like | 1106 | 2098 | 1.5446 |
| DONSON | downstream neighbor of SON | 1295 | 2332 | 1.5465 |
| SPCS3 | signal peptidase complex subunit 3 homolog | 1228 | 2210 | 1.5497 |
| TARDBP | TAR DNA binding protein | 5454 | 8759 | 1.5520 |
| CDC20 | cell division cycle 20 homolog | 4032 | 7062 | 1.5529 |
| PPIH | peptidylprolyl isomerase H | 3747 | 6571 | 1.5535 |
| LAMA3 | laminin, alpha 3 | 789 | 1636 | 1.5556 |
| LBR | lamin B receptor | 2136 | 3997 | 1.5593 |
| GTF3C3 | general transcription factor IIIC, polypeptide 3 | 943 | 1764 | 1.5605 |
| CENPF | centromere protein F, 350/400kDa (mitosin) | 2288 | 4121 | 1.5618 |
| MRE11A | MRE11 meiotic recombination 11 homolog A | 1189 | 2261 | 1.5624 |
| FN1 | fibronectin 1 | 4964 | 7994 | 1.5636 |
| IL1B | interleukin 1, beta | 3006 | 5286 | 1.5637 |
| SLC38A9 | solute carrier family 38, member 9 | 980 | 1850 | 1.5666 |
| ZWILCH | Zwilch, kinetochore associated, homolog (Drosophila) | 944 | 1802 | 1.5676 |
| ARL6IP1 | ADP-ribosylation factor-like 6 interacting protein 1 | 1779 | 3476 | 1.5697 |
| PRC1 | protein regulator of cytokinesis 1 | 1546 | 2920 | 1.5735 |
| GLG1 | golgi glycoprotein 1 | 4245 | 7156 | 1.5740 |
| BUB3 | budding uninhibited by benzimidazoles 3 homolog | 9898 | 15956 | 1.5774 |
| TIMELESS | timeless homolog | 2630 | 4776 | 1.5803 |
| ITGAV | integrin, alpha V (vitronectin receptor, alpha polypeptide, antigen CD51) | 897 | 1971 | 1.5816 |
| C15orf23 | chromosome 15 open reading frame 23 | 1250 | 2258 | 1.5835 |
| ABHD10 | abhydrolase domain containing 10 | 1247 | 2315 | 1.5880 |
| SPAG5 | sperm associated antigen 5 | 1575 | 3084 | 1.5904 |
| RRM1 | ribonucleotide reductase M1 | 3273 | 5976 | 1.5905 |
| SGOL2 | shugoshin-like 2 | 1263 | 2321 | 1.5928 |
| HIST1H4C | histone cluster 1, H4c | 13978 | 23063 | 1.5932 |
| CDCA3 | cell division cycle associated 3 | 1648 | 2978 | 1.5959 |
| AGPAT9 | 1-acylglycerol-3-phosphate O-acyltransferase 9 | 1603 | 2853 | 1.5964 |
| NSMCE4A | non-SMC element 4 homolog A | 1332 | 2397 | 1.5982 |
| RAD21 | RAD21 homolog | 3703 | 6546 | 1.5992 |
| CHAF1A | chromatin assembly factor 1, subunit A | 1207 | 2312 | 1.6024 |
| UBE2T | ubiquitin-conjugating enzyme E2T (putative) | 3774 | 7018 | 1.6126 |
| CDKN2C | cyclin-dependent kinase inhibitor 2C (p18, inhibits CDK4) | 2580 | 4849 | 1.6262 |
| KIF1B | kinesin family member 1B | 911 | 1794 | 1.6272 |
| TIMP1 | TIMP metallopeptidase inhibitor 1 | 7919 | 13714 | 1.6356 |
| LPXN | leupaxin | 1683 | 3194 | 1.6377 |
| STMN1 | stathmin 1 | 2141 | 4166 | 1.6384 |
| SASS6 | spindle assembly 6 homolog | 787 | 1592 | 1.6395 |
| POLA2 | polymerase (DNA directed), alpha 2 | 908 | 1790 | 1.6405 |
| LHX1 | LIM homeobox 1 | 2370 | 4410 | 1.6436 |
| TPX2 | TPX2, microtubule-associated, homolog | 2446 | 4820 | 1.6474 |
| TGFBI | transforming growth factor, beta-induced, 68kDa | 12156 | 20447 | 1.650 |
| TK1 | thymidine kinase 1, soluble | 3290 | 6497 | 1.6545 |
| CSRP2 | cysteine and glycine-rich protein 2 | 723 | 1643 | 1.6565 |
| WDR34 | WD repeat domain 34 | 2775 | 5236 | 1.6593 |
| CDKN3 | cyclin-dependent kinase inhibitor 3 | 6237 | 11325 | 1.6611 |
| TMEM209 | transmembrane protein 209 | 930 | 1899 | 1.6619 |
| UHRF1 | ubiquitin-like with PHD and ring finger domains 1 | 1386 | 2891 | 1.6636 |
| SDC1 | syndecan 1 | 809 | 1543 | 1.6698 |
| ASPM | asp (abnormal spindle) homolog | 2364 | 4716 | 1.6726 |
| CCDC34 | coiled-coil domain containing 34 | 1071 | 2192 | 1.6760 |
| TRIP13 | thyroid hormone receptor interactor 13 | 1719 | 3209 | 1.6805 |
| MND1 | meiotic nuclear divisions 1 homolog | 1015 | 2162 | 1.6902 |
| SMARCA2 | SWI/SNF related, matrix associated, actin dependent regulator of chromatin, subfamily a, member 2 | 4149 | 7221 | 1.6925 |
| GMPS | guanine monphosphate synthetase | 4808 | 8268 | 1.6935 |
| CCDC99 | coiled-coil domain containing 99 | 2951 | 6063 | 1.7085 |
| SH3KBP1 | SH3-domain kinase binding protein 1 | 1635 | 3457 | 1.7412 |
| GPSM2 | G-protein signaling modulator 2 | 1409 | 2978 | 1.7459 |
| DNER | delta/notch-like EGF repeat containing | 1697 | 3918 | 1.7588 |
| GJA9 | gap junction protein, alpha 9, 59kDa | 978 | 2018 | 1.7682 |
| TYMS | thymidylate synthetase | 3999 | 7858 | 1.7746 |
| ANLN | anillin, actin binding protein | 1496 | 2930 | 1.7778 |
| TNC | tenascin C | 2122 | 4124 | 1.7791 |
| RNASEH2A | ribonuclease H2, subunit A | 1857 | 3632 | 1.7822 |
| SERPINH1 | serpin peptidase inhibitor | 2032 | 4510 | 1.7933 |
| SERPINA1 | serpin peptidase inhibitor, clade A | 1989 | 4431 | 1.7989 |
| CKS2 | CDC28 protein kinase regulatory subunit 2 | 5353 | 10665 | 1.8109 |
| KIF2C | kinesin family member 2C | 2786 | 5645 | 1.8179 |
| KPNA2 | karyopherin alpha 2 (RAG cohort 1, importin alpha 1) | 7751 | 15091 | 1.8212 |
| ATAD2 | ATPase family, AAA domain containing 2 | 1397 | 3096 | 1.8408 |
| CNBP | CCHC-type zinc finger, nucleic acid binding protein | 5928 | 10906 | 1.8451 |
| DTL | denticleless homolog | 844 | 1762 | 1.8459 |
| HMGB2 | high mobility group box 2 | 6328 | 12535 | 1.8516 |
| KIF20A | kinesin family member 20A | 2256 | 4447 | 1.8577 |
| DUSP6 | dual specificity phosphatase 6 | 873 | 1795 | 1.8664 |
| MEST | mesoderm specific transcript homolog (mouse) | 893 | 1939 | 1.8695 |
| PSRC1 | proline/serine-rich coiled-coil 1 | 862 | 1939 | 1.8702 |
| AURKA | aurora kinase A | 4434 | 9087 | 1.8762 |
| MALL | mal, T-cell differentiation protein-like | 4035 | 8256 | 1.8836 |
| FAM111A | family with sequence similarity 111, member A | 1943 | 4286 | 1.8875 |
| RAD51AP1 | RAD51 associated protein 1 | 855 | 1913 | 1.8881 |
| TOP2A | topoisomerase (DNA) II alpha 170kDa | 5100 | 10228 | 1.892 |
| BORA | bora, aurora kinase A activator | 756 | 1647 | 1.8974 |
| MBOAT2 | membrane bound O-acyltransferase domain containing 2 | 732 | 1568 | 1.9079 |
| CDK1 | cyclin-dependent kinase 1 | 1243 | 2762 | 1.9387 |
| PBK | PDZ binding kinase | 948 | 2507 | 1.9568 |
| CDCA8 | cell division cycle associated 8 | 1659 | 3667 | 1.9595 |
| AURKB | aurora kinase B | 2862 | 6122 | 1.9617 |
| LAMC2 | laminin, gamma 2 | 1077 | 2652 | 1.9649 |
| NEK2 | NIMA (never in mitosis gene a)-related kinase 2 | 1036 | 2331 | 1.9704 |
| ANPEP | alanyl (membrane) aminopeptidase | 2343 | 5755 | 1.9874 |
| CCNF | cyclin F | 963 | 2156 | 2.0019 |
| KIF23 | kinesin family member 23 | 1755 | 3834 | 2.0025 |
| EXO1 | exonuclease 1 | 636 | 1559 | 2.0218 |
| FBXO5 | F-box protein 5 | 971 | 2495 | 2.0285 |
| ASPM | asp (abnormal spindle) homolog | 1256 | 3147 | 2.0369 |
| PRIM1 | primase, DNA, polypeptide 1 (49kDa) | 917 | 2063 | 2.0589 |
| BUB1 | budding uninhibited by benzimidazoles 1 homolog | 816 | 2091 | 2.0801 |
| CEP55 | centrosomal protein 55kDa | 816 | 1945 | 2.1290 |
| ITGA2 | integrin, alpha 2 (CD49B, alpha 2 subunit of VLA-2 receptor) | 1206 | 3477 | 2.1482 |
| MCM4 | minichromosome maintenance complex component 4 | 827 | 1914 | 2.1653 |
| ARHGDIB | Rho GDP dissociation inhibitor (GDI) beta | 596 | 1614 | 2.1717 |
| NDC80 | NDC80 kinetochore complex component homolog | 1739 | 4146 | 2.3126 |
| CDC25C | cell division cycle 25 homolog C | 645 | 1633 | 2.3364 |
| SPC25 | NDC80 kinetochore complex component | 1138 | 3163 | 2.3555 |
| RRM2 | ribonucleotide reductase M2 | 4233 | 11652 | 2.3663 |
| KIAA0101 | KIAA0101 | 1251 | 3292 | 2.4135 |
| CCNA2 | cyclin A2 | 2032 | 5484 | 2.4217 |
| NUSAP1 | nucleolar and spindle associated protein 1 | 1115 | 2844 | 2.4293 |
| FAM83D | family with sequence similarity 83, member D | 890 | 2278 | 2.4348 |
| HJURP | Holliday junction recognition protein | 2017 | 4994 | 2.4612 |
| FGFBP1 | fibroblast growth factor binding protein 1 | 621 | 1815 | 2.8358 |
| NT5E | 5'-nucleotidase, ecto (CD73) | 721 | 3227 | 4.1301 |
