# Supplementary material for: NT5E and FcGBP as key regulators of TGF-1-induced epithelial–mesenchymal transition (EMT) are associated with tumor progression and survival of patients with gallbladder cancer
Source: Cell Tissue Res. 2013 Dec 6;355(2):365–74. doi: 10.1007/s00441-013-1752-1 (PMC3921456; doi:10.1007/s00441-013-1752-1)
Supplement: Supplementary file 4 — (DOC 89 kb) [file 441_2013_1752_MOESM4_ESM.doc]

**Supplement Table 2. Underexpressed genes in TGF-β1-induced GBC-SD cells vs. those in control GBC-SD cells**

| **Associated Gene Name** | **Description** | **Cy5 Intensity (GBC)** | **Cy3 Intensity (TGF)** | **Ratio** |
| --- | --- | --- | --- | --- |
| FCGBP | Fc fragment of IgG binding protein | 2313 | 579 | 0.2055 |
| LCN2 | lipocalin 2 | 14424 | 4149 | 0.2386 |
| CEACAM7 | carcinoembryonic antigen-related cell adhesion molecule 7 | 1654 | 589 | 0.2456 |
| PDZK1IP1 | PDZK1 interacting protein 1 | 12457 | 4922 | 0.3272 |
| SAA4 | serum amyloid A4, constitutive | 2281 | 840 | 0.3577 |
| SLC39A10 | solute carrier family 39 (zinc transporter), member 10 | 3465 | 1506 | 0.3772 |
| STX16 | syntaxin 16 | 5587 | 2650 | 0.3798 |
| DUSP1 | dual specificity phosphatase 1 | 3895 | 1616 | 0.3814 |
| MMP7 | matrix metallopeptidase 7 (matrilysin, uterine) | 4616 | 2302 | 0.4081 |
| EML1 | echinoderm microtubule associated protein like 1 | 7840 | 3447 | 0.4118 |
| WISP2 | WNT1 inducible signaling pathway protein 2 | 2391 | 1144 | 0.4220 |
| ZG16B | zymogen granule protein 16 homolog B | 3795 | 2095 | 0.4454 |
| ZC3H12A | zinc finger CCCH-type containing 12A | 1960 | 949 | 0.4571 |
| CDADC1 | cytidine and dCMP deaminase domain containing 1 | 3735 | 2374 | 0.4628 |
| MATN2 | matrilin 2 | 3915 | 2282 | 0.4651 |
| CCL2 | chemokine (C-C motif) ligand 2 | 11201 | 6573 | 0.4661 |
| ABCB1 | ATP-binding cassette, sub-family B (MDR/TAP), member 1 | 3149 | 1806 | 0.4699 |
| EDN1 | endothelin 1 | 4845 | 2872 | 0.4917 |
| GADD45A | growth arrest and DNA-damage-inducible, alpha | 12401 | 6735 | 0.4948 |
| NR1H4 | nuclear receptor subfamily 1, group H, member 4 | 2273 | 1391 | 0.4951 |
| JUP | junction plakoglobin | 2592 | 1707 | 0.5108 |
| CFD | complement factor D (adipsin) | 5547 | 3471 | 0.5156 |
| RARRES3 | retinoic acid receptor responder (tazarotene induced) 3 | 3917 | 2389 | 0.5173 |
| THBS1 | thrombospondin 1 | 7236 | 4795 | 0.5195 |
| NFKBIZ | nuclear factor of kappa light polypeptide gene enhancer in B-cells inhibitor, zeta | 4272 | 2563 | 0.5201 |
| C14orf149 | chromosome 14 open reading frame 149 | 2705 | 1759 | 0.5215 |
| HSD17B2 | hydroxysteroid (17-beta) dehydrogenase 2 | 1788 | 1193 | 0.5316 |
| HLA-DPB1 | major histocompatibility complex, class II, DP beta 1 | 2013 | 1233 | 0.5344 |
| STAT2 | signal transducer and activator of transcription 2, 113kDa | 2034 | 1350 | 0.5362 |
| CX3CL1 | chemokine (C-X3-C motif) ligand 1 | 2525 | 1611 | 0.5363 |
| C4orf3 | chromosome 4 open reading frame 3 | 3473 | 2432 | 0.5412 |
| AKR1B10 | aldo-keto reductase family 1, member B10 (aldose reductase) | 8247 | 5127 | 0.5429 |
| SCNN1A | sodium channel, nonvoltage-gated 1 alpha | 6129 | 3853 | 0.5462 |
| AGT | angiotensinogen (serpin peptidase inhibitor, clade A, member 8) | 2530 | 1699 | 0.5469 |
| FTH1 | ferritin, heavy polypeptide 1 | 37215 | 20413 | 0.5598 |
| SH2D3A | SH2 domain containing 3A | 4536 | 2995 | 0.5621 |
| C15orf48 | chromosome 15 open reading frame 48 | 7145 | 4349 | 0.5622 |
| WWC1 | WW and C2 domain containing 1 | 9878 | 6174 | 0.5654 |
| TXNRD1 | thioredoxin reductase 1 | 29018 | 16802 | 0.5693 |
| PPP1R13B | protein phosphatase 1, regulatory subunit 13B | 3735 | 2430 | 0.5724 |
| PECR | peroxisomal trans-2-enoyl-CoA reductase | 2099 | 1531 | 0.5760 |
| AIF1L | allograft inflammatory factor 1-like | 4942 | 3387 | 0.5782 |
| CYR61 | cysteine-rich, angiogenic inducer, 61 | 8856 | 5224 | 0.5788 |
| PDP1 | pyruvate dehyrogenase phosphatase catalytic subunit 1 | 1785 | 1311 | 0.5810 |
| PLSCR3 | phospholipid scramblase 3 | 2192 | 1588 | 0.5830 |
| PEA15 | phosphoprotein enriched in astrocytes 15 | 2286 | 1578 | 0.5849 |
| TNS4 | tensin 4 | 7368 | 4556 | 0.5917 |
| FHDC1 | FH2 domain containing 1 | 1599 | 1162 | 0.5964 |
| GNS | glucosamine (N-acetyl)-6-sulfatase | 2458 | 1755 | 0.6009 |
| ANXA4 | annexin A4 | 16899 | 10839 | 0.6021 |
| RPL8 | ribosomal protein L8 | 47789 | 31310 | 0.6074 |
| CREG1 | cellular repressor of E1A-stimulated genes 1 | 9259 | 6356 | 0.6136 |
| CYFIP2 | cytoplasmic FMR1 interacting protein 2 | 2244 | 1647 | 0.6163 |
| CEBPB | CCAAT/enhancer binding protein (C/EBP), beta | 10461 | 7404 | 0.6168 |
| LYN | v-yes-1 Yamaguchi sarcoma viral related oncogene homolog | 2029 | 1576 | 0.6208 |
| HLA-DPA1 | major histocompatibility complex, class II, DP alpha 1 | 3684 | 2910 | 0.6208 |
| EXOC7 | exocyst complex component 7 | 2830 | 2203 | 0.6241 |
| HCFC1R1 | host cell factor C1 regulator 1 | 6228 | 4958 | 0.6264 |
| RPL14P1 | ribosomal protein L14 pseudogene 1 | 25926 | 17745 | 0.6309 |
| ECHDC2 | enoyl CoA hydratase domain containing 2 | 2273 | 1722 | 0.6313 |
| SDC3 | syndecan 3 | 2333 | 1904 | 0.6314 |
| DLGAP4 | discs, large (Drosophila) homolog-associated protein 4 | 7107 | 4645 | 0.6319 |
| ANXA3 | annexin A3 | 9270 | 6586 | 0.6337 |
| CDH1 | E-cadherin | 2016 | 1672 | 0.6344 |
| PPAP2C | phosphatidic acid phosphatase type 2C | 4544 | 2747 | 0.6385 |
| GABRE | gamma-aminobutyric acid (GABA) A receptor, epsilon | 3555 | 2675 | 0.6397 |
| PRSS23 | protease, serine, 23 | 9740 | 7426 | 0.6427 |
| MB21D2 | Mab-21 domain containing 2 | 2805 | 2334 | 0.6436 |
| RCAN1 | regulator of calcineurin 1 | 9595 | 6747 | 0.6452 |
| RP11-1280I22.1.1 | Plasma cell-induced resident endoplasmic reticulum protein | 3753 | 2918 | 0.6480 |
| DDIT3 | DNA-damage-inducible transcript 3 | 1667 | 1353 | 0.6501 |
| MED6 | mediator complex subunit 6 | 1779 | 1461 | 0.6509 |
| TOB1 | transducer of ERBB2, 1 | 4914 | 3705 | 0.6530 |
| IL11 | interleukin 11 | 5610 | 4521 | 0.6550 |
| BLVRB | biliverdin reductase B (flavin reductase (NADPH)) | 8529 | 5534 | 0.6585 |
| PSME1 | proteasome (prosome, macropain) activator subunit 1 | 9837 | 6901 | 0.6601 |
| PLK2 | polo-like kinase 2 | 10579 | 8444 | 0.6612 |
| NRXN2 | neurexin 2 | 1518 | 1207 | 0.6618 |
| MRPL33 | mitochondrial ribosomal protein L33 | 6927 | 4658 | 0.6627 |
| TPD52L1 | tumor protein D52-like 1 | 2581 | 2104 | 0.6637 |
| RFXANK | regulatory factor X-associated ankyrin-containing protein | 1565 | 1306 | 0.6658 |
