# Supplementary material for: NT5E and FcGBP as key regulators of TGF-1-induced epithelial–mesenchymal transition (EMT) are associated with tumor progression and survival of patients with gallbladder cancer
Source: Cell Tissue Res. 2013 Dec 6;355(2):365–74. doi: 10.1007/s00441-013-1752-1 (PMC3921456; doi:10.1007/s00441-013-1752-1)
Supplement: Supplementary file 5 — (DOC 29 kb) [file 441_2013_1752_MOESM5_ESM.doc]

**Supplement Table 3 Differential expression of NT5E and FcGBP proteins in gallbladder adenocarcinoma, peritumoral tissues, adenoma, polypus, and chronic cholecystitis**

| Disease | Total cases | NT5E | | | FcGBP | | |
| --- | --- | --- | --- | --- | --- | --- | --- |
|  |  | Positive cases (%) | $\chi^{2}$ | *p* value | Positive cases (%) | $\chi^{2}$ | *p* value |
| Gallbladder adenocarcinoma | 108 | 59 (54.6) |  |  | 52 (48.1) |  |  |
| Peritumoral tissues | 46 | 14 (30.4) | 7.57 | <0.01 | 35 (76.1) | 10.40 | <0.01 |
| Adenoma | 30 | 5 (16.7) | 13.61 | <0.01 | 24 (80.0) | 9.63 | <0.01 |
| Polyp | 15 | 2 (13.3) | 8.99 | <0.01 | 13 (86.7) | 7.84 | <0.01 |
| Chronic cholecystitis | 35 | 4 (11.4) | 20.02 | <0.01 | 30 (85.7) | 15.25 | <0.01 |
