# Supplementary material for: NT5E and FcGBP as key regulators of TGF-1-induced epithelial–mesenchymal transition (EMT) are associated with tumor progression and survival of patients with gallbladder cancer
Source: Cell Tissue Res. 2013 Dec 6;355(2):365–74. doi: 10.1007/s00441-013-1752-1 (PMC3921456; doi:10.1007/s00441-013-1752-1)
Supplement: Supplementary file 6 — (DOC 58 kb) [file 441_2013_1752_MOESM6_ESM.doc]

**Supplement Table 4 Association of NT5E and FcGBP expression with clinicopathological parameters of gallbladder adenocarcinoma**

| Clinicopathological features | Total cases | NT5E | | | FcGBP | | |
| --- | --- | --- | --- | --- | --- | --- | --- |
|  |  | Positive cases (%) | *χ^2^* | *p* value | Positive cases (%) | *χ^2^* | *p* value |
| Gender |  |  |  |  |  |  |  |
| Male | 24 | 14 (58.3) | 0.17 | >0.05 | 12 (50.0) | 0.04 | >0.05 |
| Female | 84 | 45 (53.8) |  |  | 40 (47.6) |  |  |
| Age(years) |  |  |  |  |  |  |  |
| ≤45 | 31 | 20 (64.5) | 1.72 | >0.05 | 16 (51.6) | 0.21 | >0.05 |
| >45 | 77 | 39 (50.7) |  |  | 36 (46.8) |  |  |
| Pathological type |  |  |  |  |  |  |  |
| Well differentiated | 36 | 13 (36.1) | 9.36 | <0.05 | 26 (72.2) | 13.45 | <0.01 |
| Moderately differentiated | 31 | 18 (58.1) |  |  | 12 (38.7) |  |  |
| Poorly differentiated | 30 | 22 (73.3) |  |  | 9 (30.0) |  |  |
| Mucinous adenocarcinoma | 11 | 6 (54.5) |  |  | 5 (45.5) |  |  |
| Maximal diameter of lesion (cm) |  |  |  |  |  |  |  |
| <2 | 31 | 12 (38.7) | 4.47 | <0.05 | 21 (67.7) | 6.69 | <0.05 |
| ≥2 | 77 | 47 (61.0) |  |  | 31 (40.3) |  |  |
| Lymph node metastasis |  |  |  |  |  |  |  |
| No | 49 | 17 (34.7) | 14.38 | <0.01 | 32 (65.3) | 10.58 | <0.01 |
| Yes | 59 | 42 (71.2) |  |  | 20 (33.9) |  |  |
| Invasion into the surrounding tissue |  |  |  |  |  |  |  |
| No | 49 | 18 (36.7) | 11.59 | <0.01 | 31 (63.3) | 8.21 | <0.01 |
| Yes | 59 | 41 (69.5) |  |  | 21 (35.6) |  |  |
| Gallstones |  |  |  |  |  |  |  |
| No | 50 | 25 (50.0) | 0.81 | >0.05 | 26 (52.0) | 0.55 | >0.05 |
| Yes | 58 | 34 (58.6) |  |  | 26 (44.8) |  |  |
