# Supplementary material for: NT5E and FcGBP as key regulators of TGF-1-induced epithelial–mesenchymal transition (EMT) are associated with tumor progression and survival of patients with gallbladder cancer
Source: Cell Tissue Res. 2013 Dec 6;355(2):365–74. doi: 10.1007/s00441-013-1752-1 (PMC3921456; doi:10.1007/s00441-013-1752-1)
Supplement: Supplementary file 7 — (DOC 33 kb) [file 441_2013_1752_MOESM7_ESM.doc]

**Supplement Table 5 Univariate analysis of overall survival of patients with surgically resected gallbladder adenocarcinoma**

| Clinicopathologic characteristics Cases (N) Average survival (ranges, months) *p* value |
| --- |
| Gender  Male 19 10.0 (4-16) 0.910  Female    48 10.0 (4-18)  Age (years)  ≤45 11 8.0 (4-14) 0.121  >45 56 10.0 (4-18)  Pathological type  Adenoma cancerous  8   12.0 (8-18)  Well differentiated 20 10.0 (4-18)  Moderately differentiated   20 10.0 (4-18) 0.031  Poorly differentiated   12     8.0 (4-10)  Mucinous adenocarcinoma 7   10.0 (6-16)  Maximal diameter of mass(cm)  <2 20 14.0 (4-18) 0.003  ≥2 47 8.0(4-18)  Metastasis of lymph node  No   36 12.0 (4-18) 0.005  Yes    31 8.0 (4-18)  Surrounding tissue invasion  No 39 10.0 (4-18) 0.002  Yes   28 8.0 (4-16)  NT5E  Positive 35 9.3 (4-18) 0.036  Negative 32   11.6 (4-18)  FcGBP  Positive 33   11.8 (4-18) 0.006  Negative   9.0 (4-18)  NT5E+FcGBP*  NT5E (+)/FcGBP (+) 11 10.4 (4-18) 0.039  NT5E (+)/FcGBP (－) 24 8.7 (4-18)  NT5E (－)/FcGBP (+) 22 12.3 (6-18)  NT5E (－)/FcGBP (－) 10 10.4 (6-16) |
