# Supplementary material for: NT5E and FcGBP as key regulators of TGF-1-induced epithelial–mesenchymal transition (EMT) are associated with tumor progression and survival of patients with gallbladder cancer
Source: Cell Tissue Res. 2013 Dec 6;355(2):365–74. doi: 10.1007/s00441-013-1752-1 (PMC3921456; doi:10.1007/s00441-013-1752-1)
Supplement: Supplementary file 8 — (DOC 29 kb) [file 441_2013_1752_MOESM8_ESM.doc]

**Supplement Table 6** Multivariate Cox regression analysis of overall survival of patients with surgically resected gallbladder carcinoma

| Group Category B SE (B) Exp (B) P | 95% CI for Exp (B) |
| --- | --- |
|  | Inferior Superior |
| Pathology type: Adenoma cancerous/well/moderately 0.611 0.334 1.842 0.067 0.957 3.545  /poorly differentiated/mucous carcinoma  Tumor diameter: <2.0 cm /2.0 cm 1.006 0.383 2.735 0.009 1.291 5.793  Lymphonode metastasis: No/yes 1.181 0.407 3.258 0.004 1.467 7.233  Surrounding tissue invasion: No/yes 0.114 0.364 3.047 0.002 1.493 6.218  Operation: Radical/ palliative 1.398 0.451 4.047 0.002  1.672   9.796  NT5E expression: -/+   0.936 0.399 2.550 0.019   1.166  5.574  FcGBP expression: -/+   -1.185 0.412 0.306 0.004   0.136   0.686 | |
