# Supplementary material for: NT5E and FcGBP as key regulators of TGF-1-induced epithelial–mesenchymal transition (EMT) are associated with tumor progression and survival of patients with gallbladder cancer
Source: Cell Tissue Res. 2013 Dec 6;355(2):365–74. doi: 10.1007/s00441-013-1752-1 (PMC3921456; doi:10.1007/s00441-013-1752-1)
Supplement: Supplementary file 9 — (DOC 32 kb) [file 441_2013_1752_MOESM9_ESM.doc]

**Supplement Table 1-3** GO analysis: Biological process of the genes

| **GO Term** | **Count** | **p-Value** | **q-Value** |
| --- | --- | --- | --- |
| GO:0007049 cell cycle | 40 | 2.21E-61 | 3.70E-60 |
| GO:0007067 mitosis | 30 | 2.96E-60 | 3.97E-59 |
| GO:0051301 cell division | 27 | 1.48E-51 | 1.65E-50 |
| GO:0006260 DNA replication | 17 | 1.61E-29 | 1.35E-28 |
| GO:0006974 response to DNA damage stimulus | 17 | 2.24E-27 | 1.67E-26 |
| GO:0006281 DNA repair | 13 | 5.21E-21 | 2.69E-20 |
| GO:0006355 regulation of transcription, DNA-dependent | 22 | 4.62E-19 | 1.94E-18 |
| GO:0007155 cell adhesion | 15 | 5.33E-18 | 1.98E-17 |
| GO:0048015 phosphoinositide-mediated signaling | 9 | 7.60E-17 | 2.43E-16 |
