# Supplementary material for: NT5E and FcGBP as key regulators of TGF-1-induced epithelial–mesenchymal transition (EMT) are associated with tumor progression and survival of patients with gallbladder cancer
Source: Cell Tissue Res. 2013 Dec 6;355(2):365–74. doi: 10.1007/s00441-013-1752-1 (PMC3921456; doi:10.1007/s00441-013-1752-1)
Supplement: Supplementary file 10 — (DOC 32 kb) [file 441_2013_1752_MOESM10_ESM.doc]

**Supplement Table 1-4** GO analysis: Cellular component of the genes

| **GO Term** | **Count** | **p-Value** | **q-Value** |
| --- | --- | --- | --- |
| GO:0005634 nucleus | 95 | 2.88E-112 | 1.93E-110 |
| GO:0005737 cytoplasm | 74 | 8.79E-68 | 1.96E-66 |
| GO:0005576 extracellular region | 27 | 6.93E-27 | 4.22E-26 |
| GO:0005654 nucleoplasm | 20 | 9.19E-27 | 5.13E-26 |
| GO:0005829 cytosol | 17 | 1.09E-19 | 4.86E-19 |
| GO:0000777 condensed chromosome kinetochore | 8 | 1.68E-18 | 6.61E-18 |
| GO:0016021 integral to membrane | 28 | 2.10E-17 | 7.41E-17 |
| GO:0005730 nucleolus | 13 | 3.90E-17 | 1.31E-16 |
| GO:0000775 chromosome, pericentric region | 8 | 1.50E-15 | 4.19E-15 |
