# Supplementary material for: NT5E and FcGBP as key regulators of TGF-1-induced epithelial–mesenchymal transition (EMT) are associated with tumor progression and survival of patients with gallbladder cancer
Source: Cell Tissue Res. 2013 Dec 6;355(2):365–74. doi: 10.1007/s00441-013-1752-1 (PMC3921456; doi:10.1007/s00441-013-1752-1)
Supplement: Supplementary file 11 — (DOC 32 kb) [file 441_2013_1752_MOESM11_ESM.doc]

**Supplement Table 1-5 GO analysis: Molecular function** of the genes

| **GO Term** | **Count** | **p-Value** | **q-Value** |
| --- | --- | --- | --- |
| GO:0005515 protein binding | 91 | 3.78E-91 | 1.27E-89 |
| GO:0005524 ATP binding | 26 | 1.96E-30 | 1.87E-29 |
| GO:0000166 nucleotide binding | 27 | 3.97E-27 | 2.66E-26 |
| GO:0005509 calcium ion binding | 17 | 6.95E-20 | 3.33E-19 |
| GO:0003677 DNA binding | 20 | 8.94E-17 | 2.72E-16 |
| GO:0003700 transcription factor activity | 15 | 9.47E-17 | 2.76E-16 |
| GO:0008270 zinc ion binding | 18 | 1.90E-14 | 4.91E-14 |
| GO:0016787 hydrolase activity | 17 | 1.43E-13 | 3.54E-13 |
| GO:0016740 transferase activity | 14 | 5.57E-12 | 1.17E-11 |
